# Effects of Irregular Mealtimes on Social and Eating Jet Lags among Japanese College Students

**DOI:** 10.3390/nu15092128

**Published:** 2023-04-28

**Authors:** Kazuki Nishimura, Yutaro Tamari, Yuka Nose, Hidetaka Yamaguchi, Sho Onodera, Koji Nagasaki

**Affiliations:** 1 Department of Global Environment Studies, Hiroshima Institute of Technology, Hiroshima 731-5193, Japan; 2 Department of Clinical Engineering, Hiroshima Institute of Technology, Hiroshima 731-5193, Japan; y.tamari.nf@cc.it-hiroshima.ac.jp; 3 Department of Nutritional Sciences, Yasuda Women’s University, Hiroshima 731-0153, Japan; nose@yasuda-u.ac.jp; 4 Department of Sports Social Management, Kibi International University, Okayama 716-0018, Japan; hide@kiui.ac.jp; 5 Department of Health and Sports Science, Kawasaki University of Medical Welfare, Okayama 701-0193, Japan; shote@mw.kawasaki-m.ac.jp; 6 Department of Food Sciences and Biotechnology, Hiroshima Institute of Technology, Hiroshima 731-5193, Japan; k.nagasaki.8h@cc.it-hiroshima.ac.jp

**Keywords:** social jet lag, eating jet lag, non-eating duration, midpoint of non-eating duration, chronotype, irregularities, male college students, mealtime

## Abstract

College students’ social and eating jet lags and chronotypes may be related to irregular eating habits. Therefore, we examined the relationship between social and eating jet lags, chronotypes, variability in first and last mealtimes, and non-eating duration, as well as the effects of snacking between dinner and bedtime on social and eating jet lags, chronotypes, and mealtime variation. A total of 1900 Japanese male college students were recruited in this study. Mean wake-up time, bedtime, sleeping time, first and last mealtimes, snacks between meals, non-eating duration, the midpoint of non-eating duration, social and eating jet lags, and chronotype were calculated. Standard deviations in first and last mealtimes, the midpoint of non-eating duration, and the coefficient of variation in non-eating duration were used to evaluate mealtime variations. Mealtime variations were significantly associated with social and eating jet lags, chronotype, the midpoint of non-eating duration, and the difference in first and last mealtime between school holidays and class days. Chronotype and the midpoint of non-eating duration were significantly delayed with increased snacking after dinner. Mealtime variations were significantly lower in those who avoided snacking than in those who did not. Thus, social and eating jet lags and chronotypes are associated with sleep habits and mealtime irregularities.

## 1. Introduction

Public health problems particular to college students include low-quality lifestyle habits and poor mental health [1,2,3]. One reason for this is their unique daily schedules. As college students have varying start times for each school day, they are less influenced by the social entrainment factors of circadian rhythms, such as irregular sleep and mealtimes, later chronotypes, and pronounced social jet lag [4]. Problems associated with nocturnal thinking include skipping breakfast; working late at night; short sleeping hours; and irregular times of getting out of bed, going to bed, and mealtimes. However, as the influence of social conformity factors is small, waking up late on weekdays does not necessarily indicate a high index of social jet lag [4]. The discrepancy between an individual’s biological clock and social time is called social jet lag, and pronounced social jet lag is associated with physical and mental disorders [5]. The indexes reflecting sleep habit irregularities, such as the standard deviations (SD) in times of getting out of bed, going to bed, and the coefficient of variation (CV) of time sleeping, are significantly positively correlated with social jet lag [6]. Additionally, social jet lag is reflected by differences in sleep habits between weekdays and weekends, and multiple regression analysis is performed to clarify the degree of impact of sleep indexes on social jet lag. The SD of the time of getting out of bed had the highest standardized partial regression coefficient, followed by the difference in time of going to bed during school holidays and on class days, and the difference in time sleeping on school holidays and on class days. A stepwise selection analysis adopted the SD in time of getting out of bed as the first factor [4]. These findings indicate that social jet lag is associated with an irregular time of getting out of bed and differences in sleep habits between school holidays and class days. Moreover, social jet lag is affected more by irregularities in time of getting out of bed than by prolonged sleeping time on school holidays [4].

Social jet lag is calculated as the difference between the midpoints of sleeping time on weekdays and free days [5]. It has been linked to unhealthy behaviors, such as smoking [5] and physical inactivity [7], and has been suggested as a risk factor for obesity [8] or metabolic dysfunction, which may lead to a predisposition toward diabetes, atherosclerotic cardiovascular disease [9,10,11], and depression [12]. In Japan (average age: 45.1 ± 13.4), the absolute social jet lag was 0.91 ± 0.89 h [13]. Moreover, the absolute social jet lag of those in their 20s was higher than that of the other age groups [13]. The absolute social jet lag in Japanese college students was 1.1 ± 1.0 h [4]. In Spain and Mexico, the social jet lag of university students was reported as 1.7 ± 1.0 h [14]. Based on the United Kingdom National Diet and Nutrition Survey, the social jet lag of participants who sleep less than 7 h and 7–8 h was 36 min and 12 min, respectively [15].

An individual’s biological clock is also influenced by eating and sleep habits. An American Heart Association statement pointed out that irregular eating habits, such as inconsistent timing and frequency of meals daily, can be detrimental to weight and cardiometabolic status [16]. A delay in mealtimes has been associated with decreased diet-induced thermogenesis and basal energy expenditure and impaired glucose tolerance [17,18,19,20]. Additionally, irregular mealtimes can dampen circadian rhythms, particularly those involved in anticipatory responses to feeding [21]. This predictive response to feeding monitors the regulation of nutrient homeostasis during the postprandial period [21]. However, when feeding occurs at an unusual (or unexpected) time, nutrient-sensing pathways act on the peripheral clock, and food is expected at a new time the subsequent day [21,22]. When feeding occurs at a normal (or expected) time, the circadian clock ensures that the appropriate pathways that help nutrient assimilation are activated in anticipation of feeding. Thus, organisms can handle nutrient utilization, thereby maintaining homeostasis [21]. Furthermore, consistently spaced mealtimes may maintain optimal nutrient utilization and promote health [21]. Therefore, feeding can independently activate nutrient-sensing pathways, thereby compromising food processing during the postprandial period.

Irregular mealtimes were strongly associated with a lower number of chewing cycles, shorter eating duration for each meal, eating a big meal at each mealtime, eating out frequently, having an unbalanced diet frequently, eating salty food frequently, and low vegetable intake [23]. Furthermore, it was associated with a lower frequency of breakfast consumption, higher nighttime snacking frequency, a shorter period between the last meal and sleep onset, a lower ratio of breakfast consumption, and a higher ratio of snacks consumed [23]. Although variability in meal timing has not been extensively examined, “eating jet lag” has been assessed as the difference between mealtimes on weekdays and weekends [2,14]. At the University of Barcelona (Barcelona, Spain), the absolute eating jet lag was 0.9 ± 0.7 h [2].

In addition, there was a positive association between eating-related jet lag and body mass index (BMI), independent of chronotype and social jet lag [14]. The threshold for eating jet lag was ≥3.5 h, which could significantly increase BMI [14]. Eating and social jet lag may be attributed not only to the differences in mealtimes between weekdays and weekends, but also to irregularities in mealtimes. Late mealtimes after waking up, such as by skipping breakfast, may be associated with eating jet lag. Furthermore, on some weekdays, breakfast is eaten, and on others, it is not. Irregularities in the time of the first meal of the day and/or the time between the last meal of the day and first meal of the next day may also be associated with eating jet lag. College students with night-type chronotypes eat between meals, after dinner, and at bedtime. Thus, we speculate that an increase in the frequency of snacking between dinner and bedtime may make social and eating jet lags more pronounced.

We hypothesized that college students’ social and eating jet lags and chronotypes are related to the effects of irregularities in eating habits, such as late mealtimes, the SD in mealtimes, the CV in non-eating duration, and the frequency of snacking between dinner and bedtime. Appropriate eating, sleeping, and exercising habits in college life positively affect work productivity, quality of life, and healthy life expectancy later in life. Therefore, it is important to clarify the relationship between lifestyle habits, circadian rhythms, and social and eating jet lags in male college students.

This study aimed to evaluate whether social and eating jet lags are associated with late mealtimes and irregularities in mealtimes among Japanese male college students. Therefore, we examined (1) the relationship between social and eating jet lags, chronotypes, first and last mealtimes, the SD in the first and last mealtimes, and the midpoint of non-eating duration, and the CV in non-eating duration; and (2) the effects of the frequency of snacking between dinner and bedtime on social and eating jet lags, chronotypes, mealtime, and mealtime variations.

## 2. Materials and Methods

### 2.1. Participants

#### 2.1.1. Recruitment

The study participants were Japanese male college students enrolled in the Institute of Technology and taking liberal arts courses in health and sports. Those majoring in engineering, applied information science, environmental studies, and life sciences were also included. To exclude the effects of aging and sex differences, only male college students aged 19–22 years were recruited. At the college where the participants were enrolled, the 1st period started at 8:50 a.m.; the 2nd started at 10:30 a.m.; the 3rd started at 1:15 p.m.; the 4th started at 3:10 p.m.; and the 5th started at 5:05 p.m. The number of classes and class start and end times differed among participants.

#### 2.1.2. Analysis Participants

A total of 1900 Japanese male students in college volunteered to participate in this study. The participants completed sleep and meal diaries during the eight-day period [4,6,24], which included records of their time of getting out of bed, going to bed, sleeping, breakfast, lunch, and dinner, snacks between meals, and non-eating duration. The inclusion criteria were as follows: all of the participants who provided data spanning two school holidays and five class days in sleep and meal diaries and completed the Munich Chronotype Questionnaire in the Japanese version [25] on the last day of completing the sleep and meal diaries were included. The exclusion criteria included participants with non-response days in their sleep and meal diaries, participants with invalid responses, and those who reported using alarm clocks on school holidays [4]. No participants had non-response days. Subsequently, 1703 male students were included in the final sample.

The study protocol was developed in compliance with the principles of the Declaration of Helsinki. The Ethics Committee of the Hiroshima Institute of Technology approved our study procedures (approval no. 15-003). All participants were briefed on the benefits and risks of this study, and they provided written informed consent prior to participation.

### 2.2. Data Collection

The mean time of getting out of bed, going to bed, sleeping, the first and last meal of the day, the number and timing of snacks between meals, the non-eating duration (duration between the last meal of the day and the first meal of the next day), the midpoint of non-eating duration, social and eating jet lags, and chronotype were calculated. The mealtime variability parameters used were the SD in the first and last mealtimes, the midpoint of non-eating duration, and the CV in non-eating duration. This assessment included two school holidays and five class days.

Relative social jet lag was calculated as follows [4,6,24]:

Relative social jet lag (h) = mean midpoint of sleeping time on school holidays − mean midpoint of sleeping time on class days

School holidays were defined as nights before school holidays (Friday and Saturday night), whereas class days were defined as nights before a class day (Sunday to Thursday night).

The relative eating jet lag was calculated as [2,14]

Relative eating jet lag (h) = mean midpoint of non-eating duration on school holidays − mean midpoint of non-eating duration on class days

We examined the absolute social and eating jet lags. Chronotype was calculated as the mean midpoint of sleeping time on school holidays (MSF). If the period of sleeping was longer on school holidays than on class days, the sleep-corrected MSF (MSFsc) was used as a parameter of chronotype [8].

### 2.3. Groups According to Frequency of Snacking after Dinner

The participants were classified into three groups: the skipping (no snacking after dinner), low-frequency (snacking one to three times per week after dinner), and high-frequency groups (snacking four to seven times per week after dinner).

### 2.4. Statistical Analysis

Normality was confirmed for all variables using histograms and Q-Q plots. All parameters were expressed as mean ± standard deviation. The Statistical Package for the Social Sciences for Windows version 25 (IBM, Armonk, NY, USA) was used for all calculations. Paired *t*-tests were performed to compare the mean first and last mealtimes, midpoint of non-eating duration, and non-eating duration between school holidays and class days. The effect size (r) was calculated by changing Cohen’s D [26]. The r was rated on a three-point scale (small, r < 0.1; medium, r < 0.3; large, r < 0.5). Pearson’s correlation was used to measure mealtimes, non-eating duration, absolute social jet lag, chronotype, absolute eating jet lag, and the difference in the midpoint of non-eating duration, mealtimes, and non-eating duration between school holidays and class days. To analyze the data grouped according to the frequency of snacking after dinner, an equal variance analysis was performed first. Equal variances in the data were confirmed, and an unpaired one-way analysis of variance was performed to compare the three groups. Tukey’s test was performed for post hoc analysis. Statistical significance was set at *p* < 0.05.

## 3. Results

The mean daily frequency of meals was one (18 participants, 1.1%), two (304, 17.9%), three (1153, 67.7%), four (220, 12.9%), and five (8, 0.5%). Table 1 presents a comparison of meal parameters between school holidays and class days. The first and last mealtimes and the midpoint of non-eating duration on school holidays were significantly later than those on class days. In addition, the non-eating duration on school holidays was significantly longer than that on class days. Table 2 shows the social jet lag, chronotype, eating jet lag, the midpoint of non-eating duration, mealtimes, and non-eating duration of the participants. The MSFsc (chonotype), defined as an individual’s preference to sleep at a certain time, was 5.3 (05:18 a.m.) ± 1.5 h. The relative social jet lag was 0.9 ± 1.2 h. Furthermore, the absolute social jet lag was 1.1 ± 0.9 h. The percentages of participants with absolute social jet lag of more than 1 h and 2 h were 44.0% and 13.9%, respectively. The midpoint of non-eating duration was 3.2 (03:12 a.m.) ± 1.5 h. The difference between the midpoints of non-eating duration on school holidays and class days was 0.7 ± 1.4 h. The absolute eating jet lag was 1.1 ± 1.0 h. The percentages of participants with absolute eating jet lag of more than 1 h and 2 h were 40.4% and 16.9%, respectively. The first mealtime was 10.1 (10:06 a.m.) ± 2.2 h, the last mealtime was 20.2 (08:12 p.m.) ± 1.5 h, and the non-eating duration was 13.9 ± 2.4 h. Table 3 shows the associations between variation in meal parameters and social jet lag, chronotype, eating jet lag, the midpoint of non-eating duration, and the difference in meal parameters between school holidays and class days. The SD in the first and last mealtimes were significantly associated with social jet lag, chronotype, eating jet lag, the midpoint of non-eating duration, differences in the first and last mealtimes between school holidays and class days, and differences in non-eating duration between school holidays and class days. In particular, the correlation coefficients of SD in first mealtime and eating jet lag, the difference between the first mealtime on school holidays and class days, and the difference between non-eating duration on school holidays and class days were all greater than 0.5. In addition, the correlation coefficients of the SD in the last mealtime and the difference between the last mealtime on school holidays and class days were greater than 0.5. The SD at the midpoint of non-eating duration was significantly associated with social jet lag, chronotype, eating jet lag, the midpoint of non-eating duration, differences in first and last mealtimes between school holidays and class days, and differences in non-eating duration between school holidays and class days. In particular, the correlation coefficients of the SD of the midpoint of non-eating duration and the difference between non-eating duration on school holidays and class days were greater than 0.5.

Table 4 shows the comparison between the three groups regarding the effect of frequency of snacking after dinner on social jet lag, chronotype, eating jet lag, and midpoints of non-eating duration on school holidays and class days. The number of participants in the skipping group was 1154; low-frequency group included 444 participants; and the high-frequency group included 105 participants. The frequency of snacking after dinner was 0.7 ± 1.5 times, and excluding the skipping group, 2.4 ± 1.6 times. There were significant differences in social jet lag, chronotype, eating jet lag, and midpoints of non-eating duration on school holidays and class days among the three groups. In particular, the chronotype and midpoints of non-eating duration on school holidays and class days were significantly delayed with increased frequency of snacking after dinner. In addition, eating jet lag in the skipping group was significantly lower than that in the low-frequency group. Table 5 shows the comparison between the three groups regarding the effect of frequency of snacking after dinner on mealtime parameters and mealtime variations. There were significant differences in the last mealtime, the midpoint of non-eating duration, and the non-eating duration among the three groups. In particular, the last mealtime and midpoint of non-eating duration were significantly delayed with increased frequency of snacking after dinner. In addition, non-eating duration was significantly shorter with an increased frequency of snacking after dinner. Interestingly, there were significant differences in the SD in the first and last mealtime, the midpoint of non-eating duration, and the CV in non-eating duration among the three groups. In particular, the SD in the first mealtime and midpoint of non-eating duration in the skipping group was significantly lower than that in the low-frequency group. In addition, the SD in the last mealtime and the CV in the non-eating duration were significantly lower in the skipping group than in the low- and high-frequency groups.

## 4. Discussion

This study aimed to verify whether college students’ social and eating jet lags and chronotypes are affected by irregularities in eating habits. Our results demonstrated that mealtime variations were significantly associated with social and eating jet lags. The increased frequency of snacking after dinner caused late chronotypes and irregularities in mealtimes. These results support our hypotheses. The results also clarified that social and eating jet lags are affected not only by sleep habits, but also by irregularities in mealtimes.

The mean absolute eating jetlag was 1.1 ± 1.0 h, which is 0.2 h less than that observed in a previous study evaluating the absolute eating jet lag in Spanish and Mexican participants (mean age, 21.0 ± 2.5 years; 78% women) [14]. The percentage of participants with an absolute eating jet lag of more than 1 h was 40.4%, which is 23.6% lower than that reported in a previous study that evaluated absolute eating jet lag in Spanish and Mexican participants, who were 21.0 years old [14]. Interestingly, there was only a slight difference in MSFsc between our study (5.3 ± 1.5 h) and the previous study (5.3 ± 1.2 h). Eating jet lag was defined in a previous study as the midpoint of the eating period (eating midpoint) between weekdays and weekends [14]; however, our study defined it as the midpoint of non-eating duration, because it was easy to compare sleep parameters, such as MSFsc and CV in time of sleeping, with eating parameters, such as the midpoint of non-eating duration during school holidays and CV in non-eating duration. Eating jet lag was related not only to the social jet lag and chronotype, but also to the SD in the first and last mealtimes and the CV in non-eating duration. In particular, the correlation coefficient of eating jet lag and SD in the first mealtime was r = 0.571. College students may have been late for their first and last mealtimes and/or skipped breakfast because they start classes at different times depending on the day of the week. Alternatively, this may be attributed to some participants having their first mealtime early because of club activities on school holidays.

Our results show that mealtime variability was associated with social jet lag, chronotype, the midpoint of non-eating duration during school holidays, and eating jet lag. Interestingly, we observed that irregularities in the first mealtime were significantly associated with eating jet lag and differences in non-eating duration between school holidays and class days. These data indicate that irregularities in the first mealtime were related to eating and irregularities in the non-eating duration. Moreover, for the prevention and improvement of eating jet lag, the focus should be on establishing a consistent mealtime on class days. It had been pointed out that individual chronotypes vary in terms of the timing of different activities, including sleeping and eating [27,28]. The later chronotype is characterized by a preference for a later time of getting out of bed and/or going to bed. Therefore, a delay in mealtimes on school holidays would be in line with their circadian preference [29,30]. Consequently, the delayed time of getting out of bed would be associated with a later first mealtime on school holidays. Eating jet lag may be linked to a rising circadian asynchrony [14]. It is important to highlight that a specific timing system (food clock) tracks predictable changes in energy status (via circulating nutrients and hormonal inputs) and drives rhythmic behavior in anticipation of food availability, adjusting the phase of the peripheral clocks but not the master clock [14,17,31,32]. A previous study reported that postponing the timing of the first meal (5.5 h) resulted in the delay of both plasma glucose and PER 2 rhythms in the adipose tissue, without altering the rhythm of the master clock [33]; furthermore, it highlighted the role of mealtimes as a time-giver for peripheral clocks [33]. The regularity of the first and last mealtimes may play a role in the temporal regulation of metabolism. Food consumed within a consistent 8–12 h period appears to sustain optimal nutrient utilization and promote health [21]. A 16-week intervention in young adults showed that a reduction in the duration of the eating period from 14 h to 10–12 h and regularity in breakfast timing was related to weight loss (~1.15 kg/m^2^) [29]. Furthermore, it is plausible that when energy intake is aligned with energy expenditure and clear feeding/fasting cycles are synchronized with metabolic changes, robust circadian rhythms are maintained, and consequently, health is promoted [34]. Thus, irregularities in mealtimes may trigger a misalignment between the peripheral and central clocks. These findings indicate that regularity in schedules of mealtime should be considered in health recommendations.

There were significant differences in social jet lag, chronotype, eating jet lag, and midpoints of non-eating duration on school holidays and class days according to the frequency of snacking after dinner. These data suggest that snacking after dinner is associated with later chronotypes and significant social and eating jet lags. In addition, the chronotype showed a significant delay with an increased frequency of snacking after dinner. Interestingly, the frequency of snacking after dinner did not affect the first mealtime. Therefore, the non-eating duration was significantly shorter with an increased frequency of snacking after dinner. As a mechanism by which an increased frequency of snacking after dinner causes chronotypes to get delayed, a delay in the timing of food intake and irregular mealtimes could dampen diurnal circadian rhythms, especially those involved in the anticipatory response to feeding [21]. The circadian system comprises a master clock and a network of peripheral clocks, which are arranged in a hierarchical manner [31,34]. The master clock is located in the suprachiasmatic nucleus of the hypothalamus and regulates the main body functions, such as core body temperature, blood pressure, and sleep. By contrast, peripheral clocks are present in almost all body tissues (including the liver, pancreas, muscles, and adipose tissues) and regulate many metabolic processes, such as metabolism and glucose homeostasis [35]. The master clock coordinates behavioral rhythms, such as sleep–wake and feeding–fasting cycles, and thus organizes a sequence of physiological processes to optimize metabolism, primarily through peripheral clocks [21]. Peripheral clocks can be synchronized by the timing of food intake and can alter the internal synchrony between the master and peripheral clocks. These findings suggest that late snacking after dinner and irregularity of mealtimes may promote a night chronotype and social and eating jet lags. Snacking after dinner caused increased irregularity of mealtimes. In particular, the SD and CV of mealtimes were high for the low-frequency group. Based on these findings, it can be suggested that even if people snack after dinner about once every two days, the delay and irregularity of the time period of ingestion may cause a night chronotype, exacerbate social and eating jet lags, and increase the variation of mealtimes. It is conceivable that chronotypes and social and eating jet lags can be adequately addressed by refraining from snacking after dinner.

This study has some limitations. First, sleep and meal diaries were recorded for only eight days. The results of this study may have been influenced by the inherent variability of short recording periods. However, similar to previous studies [4,6,24,36], sleep and meal diaries in this study included two school holidays and five class days. The period covered by the diary is thought to reflect a typical week for male college students. Second, only self-reported data were collected. Nonetheless, our study has certain strengths, including its large sample size. Third, the effect of the type of meals and diet on the data was unclear because we only focused on meal timing. Therefore, it is necessary to examine in detail the content of breakfast and snacks, alcohol intake, and caffeine intake. Additionally, we did not obtain information regarding participants’ exposure time to outdoor light, screen time, physical activity, or the seasonal characteristics of light exposure. Moreover, the lifestyles of college students are presumed to be influenced by various factors, such as BMI, type of housing, school commute method, and financial situations. However, these factors were not considered in the present study. Finally, all participants were Japanese male students in college. Therefore, our conclusions might not be generalizable to the entire Japanese population or to international populations. Future studies should include participants from different age groups and sexes to corroborate the findings of the present study conclusively.

## 5. Conclusions

We examined mealtime factors affecting social and eating jet lags among Japanese male students in college. Variations in mealtimes were significantly associated with social and eating jet lags, chronotype, the midpoint of non-eating duration, and differences in first and last mealtimes between school holidays and class days. The increased frequency of snacking after dinner differed significantly according to chronotype, the midpoint of non-eating duration on school holidays and class days, and SD in the first mealtime and the midpoint of non-eating duration. Moreover, this study clarified that social and eating jet lags are affected by mealtime irregularities, such as snack intake between dinner and bedtime. These findings might contribute to the development of new programs for improving eating habits. For example, improving lifestyle habits, such as refraining from snacking after dinner and standardizing mealtimes, will eliminate social and eating jet lags, which, in turn, may reduce mental and physical disorders associated with social and eating jet lags.

## Figures and Tables

**Table 1:** Comparison of meal parameters between school holidays and class days.

	Class Days	School Holidays	*p*-Value	Effect Size	95% Confidence Interval	T-Score
First mealtime (h)	9.8 ± 2.4	11.0 ± 2.7	0.000	0.50	1.187–1.402	23.612
Last mealtime (h)	20.2 ± 1.5	20.3 ± 1.8	0.011	0.06	0.019–0.148	2.534
Midpoint of non-eating duration (h)	3.0 ± 1.5	3.7 ± 1.7	0.000	0.45	0.624–0.754	20.759
Non-eating duration (h)	13.5 ± 2.5	14.8 ± 3.0	0.000	0.43	1.090–1.332	19.690

**Table 2:** Social jet lag, chronotype, eating jet lag, midpoint of non-eating duration, and first and last mealtime and non-eating duration.

	Mean ± SD
Relative social jet lag (h)	0.9 ± 1.2
Absolute social jet lag (h)	1.1 ± 0.9
Chronotype (h)	5.3 ± 1.5
Relative eating jet lag (h)	0.7 ± 1.4
Absolute eating jet lag (h)	1.1 ± 1.0
Midpoint of non-eating duration (h)	3.2 ± 1.5
First mealtime (h)	10.1 ± 2.2
Last mealtime (h)	20.2 ± 1.5
Non-eating duration (h)	13.9 ± 2.4

Chronotype was calculated using the midpoint of sleeping time on free days (MSF). If sleep time was longer on free days than on weekdays, sleep-corrected MSF was used as an indicator of chronotype.

**Table 3:** Associations between variation in meal parameters and social jet lag, chronotype, eating jet lag, midpoint of non-eating duration, and difference in meal parameters between school holidays and class days.

	Standard Deviations in First Mealtime			Standard Deviations in Last Mealtime			Coefficient of Variation in Non-Eating Duration		
	β	95% CI	*p*-Value	β	95% CI	*p*-Value	β	95% CI	*p*-Value
Absolute social jet lag	0.408	0.368–0.447	0.000	0.146	0.099–0.192	0.000	0.276	0.232–0.319	0.000
Chronotype	0.347	0.305–0.388	0.000	0.136	0.089–0.182	0.000	0.198	0.152–0.243	0.000
Absolute eating jet lag	0.571	0.538–0.602	0.000	0.299	0.255–0.342	0.000	0.370	0.328–0.410	0.000
Midpoint of non-eating duration during school holidays	0.486	0.449–0.521	0.000	0.196	0.150–0.241	0.000	0.280	0.236–0.323	0.000
Difference in first mealtime between school holidays and class days	0.690	0.664–0.714	0.000	0.160	0.113–0.206	0.000	0.493	0.456–0.528	0.000
Difference in last mealtime between school holidays and class days	0.182	0.136–0.228	0.000	0.627	0.597–0.655	0.000	0.345	0.302–0.386	0.000
Difference in non-eating duration between school holidays and class days	0.581	0.549–0.612	0.000	0.245	0.200–0.289	0.000	0.594	0.562–0.624	0.000

β, unstandardized coefficient; CI, confidence interval.

**Table 4:** Comparison between the three groups regarding the effect of frequency of snacking after dinner on social jet lag, chronotype, eating jet lag, and midpoints of non-eating duration on school holidays and class days.

	Group			*p*-Value	F-Value
	Skipping	Low Frequency	High Frequency		
Number	1154	444	105		
Social jet lag (h)	1.1 ± 0.9	1.2 ± 1.0	1.2 ± 0.9	0.041	3.192
Chronotype (h)	5.2 ± 1.4	5.4 ± 1.5 ^a^	5.9 ± 1.5 ^a,b^	0.000	15.62
Eating jet lag (h)	1.1 ± 1.0	1.2 ± 1.1 ^a^	1.2 ± 1.1	0.019	3.954
Midpoint of non-eating duration in class days (h)	2.8 ± 1.5	3.3 ± 1.4 ^a^	3.9 ± 1.5 ^a,b^	0.000	36.892
Midpoint of non-eating duration in school holidays (h)	3.5 ± 1.6	3.9 ± 1.6 ^a^	4.6 ± 1.8 ^a,b^	0.000	35.223

Post hoc test (Tukey method), ^a^ *p* < 0.05 vs. skipping group; ^b^ *p* < 0.05 vs. low-frequency group.

**Table 5:** Comparison of the effect of frequency of snacking after dinner on mealtime parameters and mealtime variations.

	Group			*p*-Value	F-Value
	Skipping	Low Frequency	High Frequency		
Number	1154	444	105		
First mealtime (h)	10.0 ± 2.3	10.3 ± 2.1	10.3 ± 2.1	0.098	2.329
Last mealtime (h)	19.9 ± 1.3	20.6 ± 1.1 ^a^	21.9 ± 1.5 ^a,b^	0.000	135.214
Midpoint of non-eating duration (h)	3.0 ± 1.4	3.5 ± 1.3 ^a^	4.1 ± 1.4 ^a,b^	0.000	43.921
Non-eating duration (h)	14.1 ± 2.5	13.6 ± 2.0 ^a^	12.5 ± 2.2 ^a,b^	0.000	25.342
Standard deviations in first mealtime (h)	1.7 ± 1.1	1.9 ± 1.0 ^a^	1.8 ± 1.0	0.026	3.644
Standard deviations in last mealtime (h)	1.2 ± 0.9	1.7 ± 0.8 ^a^	1.5 ± 0.9 ^a^	0.000	52.738
Standard deviations in midpoint of non-eating duration (h)	1.1 ± 0.6	1.3 ± 0.6 ^a^	1.3 ± 0.7	0.000	20.798
Coefficient of variation in non-eating duration (%)	15.4 ± 8.0	18.2 ± 7.6 ^a^	18.6 ± 9.0 ^a^	0.000	23.877

Post hoc test (Tukey method), ^a^; *p* < 0.05 vs. skipping group, ^b^; *p* < 0.05 vs. low frequency group.

## Data Availability

Data supporting the findings of this study are available from the corresponding author upon request. The data are not publicly available owing to privacy and ethical restrictions.
